# Development and Calibration of an Eye-Tracking Fixation Identification Algorithm for Immersive Virtual Reality

**DOI:** 10.3390/s20174956

**Published:** 2020-09-01

**Authors:** Jose Llanes-Jurado, Javier Marín-Morales, Jaime Guixeres, Mariano Alcañiz

**Affiliations:** Instituto de Investigación e Innovación en Bioingeniería (i3B), Universitat Politècnica de València, 46022 Valencia, Spain; jamarmo@i3b.upv.es (J.M.-M.); jaiguipr@i3b.upv.es (J.G.); malcaniz@i3b.upv.es (M.A.)

**Keywords:** eye-tracking, fixation identification, virtual reality, immersive virtual reality, head-mounted display, calibration, area of interest

## Abstract

Fixation identification is an essential task in the extraction of relevant information from gaze patterns; various algorithms are used in the identification process. However, the thresholds used in the algorithms greatly affect their sensitivity. Moreover, the application of these algorithm to eye-tracking technologies integrated into head-mounted displays, where the subject’s head position is unrestricted, is still an open issue. Therefore, the adaptation of eye-tracking algorithms and their thresholds to immersive virtual reality frameworks needs to be validated. This study presents the development of a dispersion-threshold identification algorithm applied to data obtained from an eye-tracking system integrated into a head-mounted display. Rules-based criteria are proposed to calibrate the thresholds of the algorithm through different features, such as number of fixations and the percentage of points which belong to a fixation. The results show that distance-dispersion thresholds between 1–1.6° and time windows between 0.25–0.4 s are the acceptable range parameters, with 1° and 0.25 s being the optimum. The work presents a calibrated algorithm to be applied in future experiments with eye-tracking integrated into head-mounted displays and guidelines for calibrating fixation identification algorithms

## 1. Introduction

Virtual Reality (VR) is a rapidly improving emerging technology [1]. While the gaming industry is taking the lead in the development of VR, it has also found many research applications. VR allows the simulation of experiences which create the sensation of being in the real world [2]. It is very helpful in human-subject-based experiments that are difficult to perform in the real world; it offers environment simulations under controlled laboratory conditions where researchers can efficiently isolate and manipulate features while keeping the other environmental stimuli unchanged [3,4]. VR not only allows free navigation and real-world type movement [5], it can also evoke similar emotions and cognitive process to physical environments [6,7]. There are three types of VR, differentiated by the degree of immersion that the technology provides: non-immersive, semi-immersive and immersive [1]. Virtual environments displayed on single-screens, such as desktop PCs, are classified as non-immersive [8]. Powerwall screens, or cave automatic virtual environment (CAVE) technologies, achieve higher degrees of immersion [9,10]. This technological environment is classified as semi-immersive VR. Immersive virtual environments (IVE), using head-mounted display (HMD) technologies, provide the highest degree of immersion. HMDs isolate the subject from external world stimuli and provide a complete simulated experience [11]. Continuing technical HMD upgrades, such as in resolution and field of view, are increasing researcher’s interest in and use of this technology [1,12]. Technologies, such as HTC Vive or Oculus Rift, allow six degrees of freedom (DoF) inside the IVE, which is crucial for whole-room VR experiences [13], whereas other types of HMD, such as Oculus Go, have only three DoF, which is the simplest form of user tracking in VR. This is an important difference because increased DoF gives higher sense of presence inside the VR [14].

The development of VR technologies has enhanced research into understanding human behavior [1]. Moreover, in addition to classic self-assessment, VR can be combined with several implicit measures which model unconscious processes, such as electrodermal activity (EDA), heart rate variability (HRV) [7,15], and eye tracking (ET). ET is the analysis of eye movements based on corneal reflection and pupil detection. It is an important source of data for obtaining a complete dataset of features from the analysis of different types of eye movements [16]. ET studies what a subject is looking at [17,18]. During recent years, developments in ET technology have allowed it to be incorporated into many devices, such as screens, mobile ET glasses, and HMDs. ET has a powerful application in VR, as has been shown in many previous studies. For example, Tanriverdi et al. (2000) [19] measured the difference in the interaction between eye movements and hand pointing in VR scenarios to assess spatial memory. More recently, Skulmowski et al. (2014) [20], using ET, studied the psychological behavior of subjects in VR, and Juvrud et al. (2018) [21] and Clay et al. (2019) [22] highlighted and explored ET applications through immersive VR devices.

Eye movement has been studied mainly in two different experimental designs, world-centered and head-centered [23,24,25]. The principal difference between them is the origin of the coordinate system. In the world-centered design the gaze is directed at the 2D display, while the subjects is positioned in front of a screen, with restricted head movement [26]. This system is used in experimental designs where remote or desktop-integrated eye trackers are used [27]. The head-centered design, on the other hand, measures gaze points within the video recording, which moves as the subject moves his/her head. In this case, the subject can freely move during the experiment; however, the origin of the gaze coordinates is in the video display. This has big advantages in real-world experiments, but involves some difficulties in automatically identifying what the subject is looking at. This type of system derives from advanced technologies, such as mobile eye trackers (MET) [27]. However, the eye trackers integrated into HMDs present a new VR-centered framework, where the origin of the coordinate system is the virtual environment. The subject can move freely inside the VR scenario, and the impact point of the gaze is calculated using the intersection between the gaze ray and the polygons of the virtual environment.

One of the most useful methods of modeling gaze-behavior patterns is through fixation classification. A fixation is defined as a cluster of points where the distance between points is not greater than a certain value and its temporal interval is longer than a certain time. Intuitively, it has been interpreted as a group of points where a subject has focused his/her gaze [17]. There has been extensive discussion in the literature about the minimum time and dispersion distance to define a fixation. It has commonly been considered that the minimum fixation time has to be above 0.1 s [28]. The minimum time depends on the task being performed by the subject. For tasks, such as reading and visual search, the minimum fixation time stipulated is 0.225 s and 0.275 s, respectively. For tasks where eye-hand coordination is required, the mean fixation time has been established at 0.4 s [17]. In summary, mean fixation time has been established as between 0.15 s to 0.65 s [18]. The dispersion angle of fixations is not as yet defined, but they are normally fixed below $2^{\circ }$ [17]. To perform fixation classification analysis, there are three main types of spatial criteria algorithms; velocity-based, dispersion-based and area-based [29]. Velocity-based algorithms use the eye’s velocity information assuming that fixation points have low velocities and saccades points higher ones. One of the most popular algorithms is called velocity-threshold identification (I-VT). Dispersion-based algorithms emphasize the spatial distance between points using at the same time temporal and spatial information. They are based on the idea that spatial distance is lower in fixations than in saccades. The robustness and accuracy achieved is better than the two other types of dispersion based algorithms [29]. A representative algorithm from this type is the dispersion-threshold identification (I-DT). Finally, area-based algorithms identify a group of points which are inside an Area of Interest (AoI). All three types of ET fixation identification algorithms need a set of thresholds and spatial and temporal information to classify eye movements.

These fixation identification algorithms are mainly applied in world-centered and head-centered experiments. Very little work has been done on fixation classification in the VR-centered paradigm [22,30]. In world- and head-centered paradigms, only 2D gaze vectors have been studied; however, for VR-centered, ET provides two 3D vectors, gaze and head position [26,31]. How to apply both sets of vectors, in order to study eye movements, is an underaddressed challenge. Duchowski et al. (2002) [30], to obtain visual angle, proposed a solution where head position is averaged for every set of points that can be included inside a fixation. While this solution is a valuable contribution, the specification of the optimum parameters for a concrete ET fixation classification algorithm in VR-centered design remains an open question. Duchowski et al. (2002) [30] introduced the parameters of the 3D implemented algorithm, manually aligning the gaze-interaction points of subjects with the environmental targets displayed. On the other hand, it is not believed that the gaze acquisition that derives from the VR engine has an influence on eye-signal frequency, as this is influenced by the velocity of the renderization. In a later world-centered study, Bobić et al. (2016) [32] examined the number of predicted and real saccades in a guided task, using an I-VT algorithm, whereas other studies, such as Reference [24,33,34], did not report the exploration of different sets of parameters for fixation classification tasks. It is critical to identify in the related literature the best set of parameters for the algorithm, because very different results can be obtained, and interpretations made, depending on the parameters used [28,35,36]. Moreover, the methodology that should be used to identify this optimum region is still an open issue. There is no clear way to infer which is the optimum set of parameters for an ET fixation classification algorithm. For example, Blignaut (2009) [35] researched the optimum dispersion threshold for a I-DT algorithm in a free world-centered task, examining different features, and obtained an optimum region between $0.7^{\circ }$ and $1.3^{\circ }$ for radius threshold, using a time window of 0.1 s. There is still no consensus of how to achieve the optimum parameters for an ET fixation classification algorithm.

This study proposes a new methodology to calibrate a VR-centered fixation classification algorithm using ET integrated into an HMD. A guided experiment was designed to study the fixation identification of an I-DT algorithm applied to an IVE. While there is no ground truth that identifies the optimum parameters for any particular feature [35], four different features were examined in this task. A set of rules was established for each, with the aim of reaching an agreement between the features as a criterion to achieve the most suitable parameters. A final set of optimum thresholds is proposed for use with the I-DT algorithm in future research.

## 2. Materials and Methods

### 2.1. Participants

A group of 57 healthy volunteers (27 females and 30 males), with normal or corrected-to-normal vision, was recruited to participate in the experiment. The mean age of the group was 25.36 (SD = 4.97). The inclusion criteria were as follows: age between 18 and 36 years; Spanish nationality; not having any previous VR experience. All methods and experimental protocols were performed in accordance with the guidelines and regulations of the local ethics committee of the Polytechnic University of Valencia.

### 2.2. Virtual Environment and Data Collection

**Figure 1:**
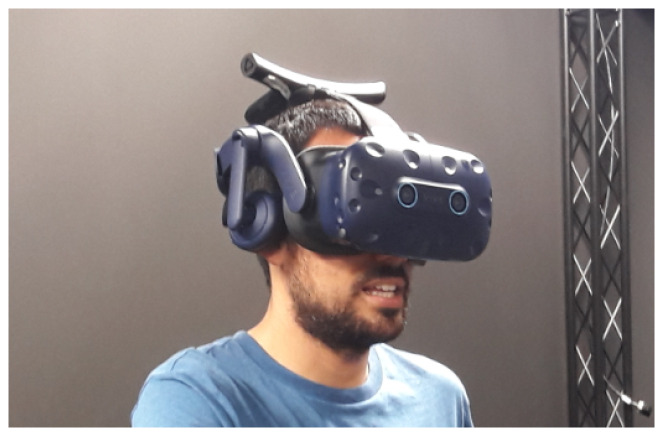
Example of a subject using the HTC Vive Pro Eye for the development of the experiment.

The virtual environment was displayed through an HTC Vive Pro Eye [37], an HMD with an integrated ET system (see Figure 1), offering a field of view of $110^{\circ }$. The scene is displayed with a resolution of 1440 × 1600 pixels per eye, with a refresh rate of 90 Hz. The set-up includes HTC Wireless Adapter, and an HTC base station covering a 6×6 m^2^ area. The ET data were obtained from the Unity VR through the ET SDK (SRanipal), with a maximum frequency of 120 Hz and an accuracy of $0.5^{\circ }$−$1.1^{\circ }$. The computer used was an Intel Core i7-770 CPU 3.60 GHz with an NVIDIA GeForce GTX 1070. To perform the study an immersive 3D scenario, using the Unity 3D platform, was developed. This features a room modeled by an occlusive Cube Map, which is a Unity object that captures all the possible stimuli on the object’s surface. This type of object, ensures that all the projections of the eye-tracker rays impact against an element in the scene to provide continuous feedback of the subject’s gaze.

**Figure 2:**
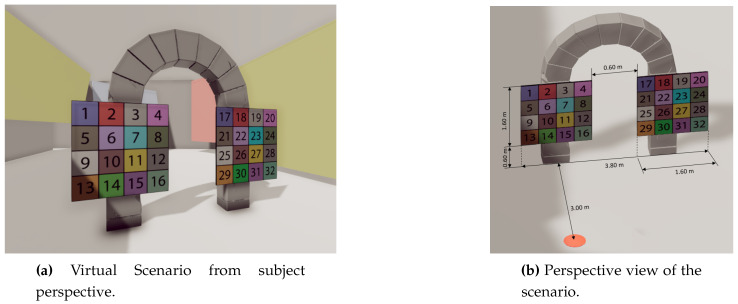
Virtual scenario screenshots. The orange dot (b) designates the position of the subject.

The room includes two large similarly-sized panels. Each panel displays a matrix of 4 × 4 numbers, where every square is identified by a sequence from 1 to 16 in the first panel, and 17 to 32 in the second. Each square includes a background color to ensure contrast between the cells and focus the subject’s attention (see Figure 2a). The initial location of the viewer is above a marked orange point in the scene, in front of one of the panels (Figure 2b). The location of this orange point was established to provide frontal gaze to one panel (from $-14.93^{\circ }$ to $14.93^{\circ }$), and diagonal gazes in the other one (from $25.02^{\circ }$ to $45.00^{\circ }$), to ensure that the subject moved his/her head during the experiment.

Several squares were lit following a pre-determined sequence designed to evoke many different fixations. The subjects were asked to look at the illuminated squares during the task. The sequence was the same for all subjects. It had been created randomly with the following guidelines: It had to begin in the front panel and explore its four diagonals and its center. Next, the subject had to look at the furthest and nearest points of the second panel (on the right). Finally, from a certain square the sequence changed, such that the squares lit alternated between the panels, first the left, then the right, etc. The resulting sequence was 1, 16, 4, 13, 6, 11, 7, 10, 17, 32, 22, 10, 20, 5, and 30. The subjects were asked to freely explore the environment for 1 min to adapt to it. After that, every square was lit for 3 s following the predetermined sequence, the total time of the guided task being 45 s. Every lit square was defined as an AoI.

The raw eye-tracking data included the 3D position of the impact of the gaze ray in the environment, and the 3D head position in the virtual space. This data is the input of the I-DT algorithm. The gaze point includes the coefficient, for each eye, of the probability that an eyelid movement constitutes a blink, where 1 means completely closed, and 0 open. Points above 0.75 in either eye were considered as blink points and removed [31]. This represented 0.69% of the total raw data. Moreover, the virtual environment exports a file, which recorded when a specific square was lit (e.g., square 1; time 0s–3 s ). This file was used to synchronize the gaze data with the illuminated sequence protocol. Only data that were, in terms of time, between the first and last lit squares were taken into account.

### 2.3. Fixation Identification Algorithm

The algorithm implemented is an adaptation of an I-DT algorithm (see the code in Supplementary Materials). A previous study suggests the use of this algorithm due to its robustness and accuracy in the fixation identification task and its low number of parameters (dispersion and time threshold) [29]. Moreover, the I-DT algorithm has been used in many previous ET parametrical studies [35,36]. In accordance with the VR-centered paradigm, the algorithm considers 3D points which are intersections of the gaze rays with virtual objects in which origin is the 3D head position. For fixation identification, head position was averaged every time that a point was added as a candidate to be a part of the fixation. Averaging the head position of the subject will take into account the free 3D movement of the subject inside the VR. Then, each beam considered as a part of a fixation will have its origin in an averaged head position [30]. It is important to note that the methodology followed or the algorithm used are not dependent on the dimensionality of the experiment. Both could be used with 2D data; however, this work examines a VR-centered experiment designed in 3D. To measure the distance between a set of points, dispersion distance (DD) [36] was used. DD measures the angular distance between the pairs of points that are candidates to be a part of a fixation. The algorithm records a set of consecutive points with time differences smaller than a specific value (line 2). The highest distance in the group has to be less than the dispersion threshold value (line 5 and 6 of Algorithm 1) to consider the set of points as a potential fixation. The dispersion threshold and time window are parameters which have to be set initially to the I-DT algorithm. Both are essential for the fixation classification task. In addition, the algorithm we present applied, as an innovation, a frequency threshold below which possible fixation points were discounted (line 3 and 6 of Algorithm 1). This ensured that the algorithm did not use gaze data recorded at frequencies that do not facilitate the detection of fast eye movements. The temporal decrease of the data collection frequency could be provoked by an increment in the graphic renderization requirements of the GPU environment or saturation of the computing capacity, which needs to be taken into account in a VR-centered framework. Since the raw ET data was obtained using the ET SDK (SRanpial) through a Unity script, the frequency of the data depends on the processing velocity of the graphic engine. Therefore, although the ET device works at 120 Hz, acquisition will be lower if the Unity rendering frequency is lower, which is highly dependent on the GPU of the computer used and the complexity of the environment. Frequency variation during the experiment was analyzed. To ensure the quality of the fixation classification, the frequency threshold was set at 30 Hz, the lowest frequency the literature uses to study ET data [31]. The pseudocode of the algorithm can be seen as follows.

**Algorithm 1:** Dispersion Algorithm

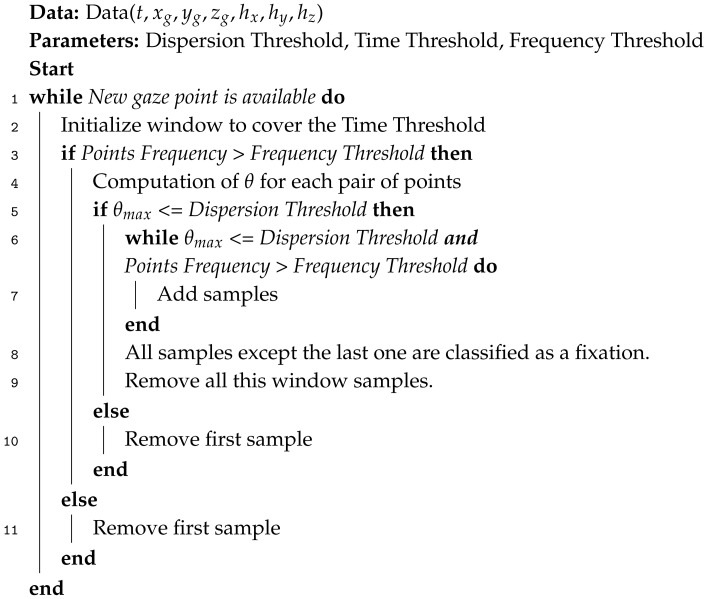



Where *t* is the time, $x_{g},y_{g},z_{g}$ are gaze coordinates, and $h_{x},h_{y},h_{z}$ are head position points. The dispersion angle θ is obtained from the scalar product between two vectors, as in Equation (1):

(1) $$\cos\theta_{ij}=\frac{{\vec{d}}_{i}\cdot {\vec{d}}_{j}}{|{\vec{d}}_{i}\left|\right|{\vec{d}}_{j}|}\;,\mathrm{with}\;{\vec{d}}_{n}={\vec{g}}_{n}-\vec{\bar{h}}\;.$$

The sub-indexes *i* and *j* are two arbitrary points, and $d_{n}$ is the final end-point of the subject’s gaze *n*, the origin of which is the average head position in each component $\vec{\bar{h}}=\left({\bar{h}}_{x},{\bar{h}}_{y},{\bar{h}}_{z}\right)$ [30].

### 2.4. Calibration Criteria

To identify suitable parameters for the I-DT algorithm a parametrical analysis examined different features. No feature exists that ensures the optimum set of parameters, or any ground truth, that can quantify how good are the parameters used by the ET algorithm [35]. An agreement between four different features was used as an appropriate criteria to evaluate the algorithm’s optimum parameters, dispersion threshold and time window. The features used in the study are averaged between all the subjects. They are discussed over the next paragraphs; a set of requirements was established. The four features used were, number of fixations, percentage of points classified as a part of a fixation, mean fixation time, and percentage of fixations inside AoIs. These features were examined in terms of the dispersion thresholds and time windows in a grid search [35,36]. The objective is to find a set of points that simultaneously satisfy the conditions imposed for each feature. This set of points would be the optimum to use for an I-DT algorithm in a VR-centered experiment. The grid used to calibrate the algorithm started at $0^{\circ }$ and went to $2.5^{\circ }$, in steps by of $0.1^{\circ }$, and time windows from 0.1 s to 0.5 s by intervals of 0.05 s. It is important to note that the first three features can be computed for guided and free tasks, whereas the fourth can be computed only for guided task protocols where specifics AoIs are defined. This calibration method used the first three features to obtain the optimum calibration, and the fourth, a specific feature which depends on the type of study, to specify and obtain the final calibration results.

- **Number of fixations** This measures the average number of fixations per subject during the task. For small dispersion thresholds the growth of the feature increases from zero until a maximum. After this maximum, the feature decreases until one single fixation for a high dispersion threshold and any time window value is obtained. The parameters to be selected must all exceed the maximum number of fixations due to the high instability in this region [35].
- **Percentage of points classified inside a fixation** measures the amount of points classified as part of a fixation. This feature is linked to the number of fixations. In the first step, with a small change in the dispersion threshold, the percentage of points increases exponentially. However, this increasing tendency changes when a maximum number of fixations is reached, which produces an elbow point in the feature [35]. After this point, the growth of the curve becomes smoother until it reaches 100% of the points included as part of a fixation. This feature helps identify a lower-limit for the dispersion threshold. Moreover, this feature has to be as high as possible to classify more points as fixation points [35]. Following this condition, this feature determines a single point and not a region of points.
- **Mean fixation time** This measures the average fixation time per subject. As has been seen in previous studies, this feature follows a linear relation with the dispersion threshold of the I-DT algorithm [35,36] in 2D world-centered experiments. The mean time fixation increases proportionally as this parameter increases. This is due to the fact that the more points there are inside a fixation, increases in the dispersion threshold involve increases in fixation time. Parameters which involve mean fixation times above certain mean times cannot be considered as optimum. This condition defines an upper-limit for the dispersion threshold and time window. In this work, the predefined maximum mean time is established at 1.5 s. Despite the fact that this fixation time is higher than the upper limit established in the literature (0.65 s [18]), it is close to the results obtained by Reference [30] in an IVE, that is, 1.9 s mean fixation time.
- **Percentage of fixations in AoI.** This measures the percentage of fixations with centers inside AoIs. A similar feature was used by Reference [30,32] to calibrate algorithms. In the present work, the majority of the fixation centers were found to be inside the defined AoIs. The percentage is obtained when the AoI is lit. This measure not only provides spatial information about where the fixation center is located, it also provides temporal information, because it only records gaze points inside an AoI when it is lit. With the variation of the parameters of the algorithm, different numbers of points are classified as part of fixations and the positions of the centers of the fixations also change. The feature starts with the highest value (close to 100%). While the dispersion threshold is increased, more points are therefore part of the same fixation. In consequence, the center of the fixation is displaced in order to be in the average position of all the fixation points. These displacements cause the center of the fixation to be placed randomly in the environment for high dispersion threshold values instead of being centered around an AoI. This evolution tends to induce a decrease in the percentage of fixations inside AoIs. However, we hypothesize that during this decrease there is a stable region where the variation of the parameters does not affect the percentage of fixations inside the AoI. This region represents the set of parameters that better model visual attention, as the values of this feature are unaffected by small changes in the parameters. The search of this stable region requires two previous steps. First, a simple moving average (SMA) of three points is used on the signal in order to smooth it and eliminate noise. After that, the first derivative of the feature is computed. Stability is considered to be established when the variation in points is below 2%. Table 1 summarizes the criteria and the features used to calibrate the ET algorithm.

Table 1 shows the criteria and the features used to calibrate an ET algorithm.

Based on the criteria in Table 1, the strategy followed in the calibration process was: (1) Computation of the features which do not depend on the definition of an AoI as being constituted by number of fixations, percentage of points classified inside a fixation and mean fixation time, (2) to compute the optimum value based on maximizing the percentage of points which belong to a fixation, (3) Step 1 is recomputed by including the percentage of fixations inside AoIs. (4) Step 2 is repeated. Therefore, to obtain the optimum set of parameters, steps 1 and 2 take account only of the free-task related features, while 3 and 4 take account of these features and the guided task related feature.

## 3. Results

### 3.1. Frequency Analysis

**Figure 3:**
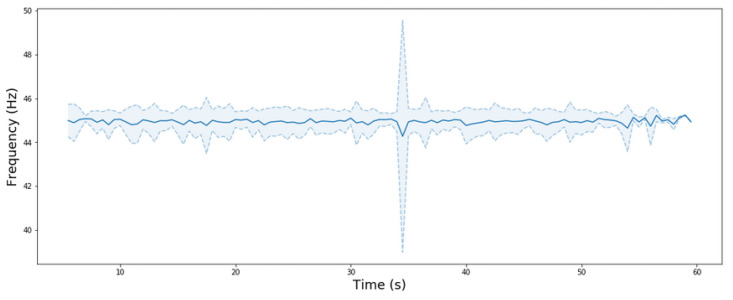
Temporal evolution of the eye tracking (ET) data frequency. The blue line is the average frequency by subject for each second. The discontinuous line indicates the standard deviation above and below the mean frequency.

The evolution of the frequency of the eye-tracking data acquisition averaged by all the subjects during the experiment is shown at Figure 3 including mean and standard deviations. The fluctuation of the frequency is mainly between 44–46 Hz where the mean frequency is 44.95 Hz. However, the ET data recording frequency of one subject was below 10 Hz between 34.5–35 s This anomalous frequency caused the high-frequency variation shown in Figure 3. The acquisition of the data mostly complies with the minimum accepted frequency established in 30 Hz.

### 3.2. Algorithm Calibration

**Figure 4:**
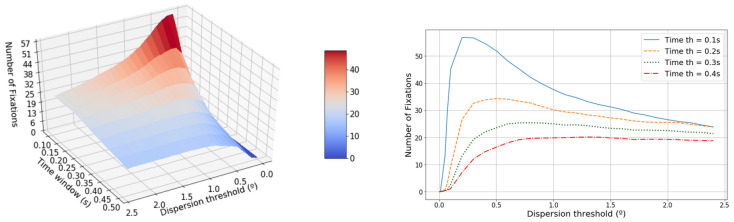
The left-hand graphic shows the evolution of the average number of fixations in terms of the dispersion threshold and the time window. The right-hand graphic shows the projected dispersion threshold.

Figure 4 shows that the number of fixations strongly depends on both parameters. Two different regions can be distinguished in this feature. The first region is defined from $0^{\circ }$ until the maximum number of fixations, between 0.25–0.6°. This region shows an increase in the number of fixations until the maximum is reached. After that, the features decreases smoothly as both parameters are higher. The highest number of fixations is achieved with the minimum time window (0.1 ms) and with a dispersion threshold of $0.25^{\circ }$. However, this maximum becomes smoother with greater time windows.

**Figure 5:**
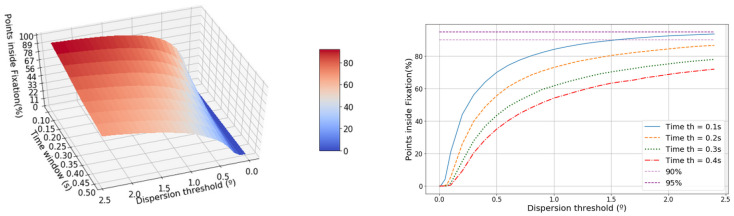
The left-hand graphic shows the evolution of the percentage of points classified as a part of a fixation in terms of the dispersion threshold and the time window. The right-hand graphic shows the projected dispersion threshold.

The evolution of the percentage of points classified as fixation points is shown in Figure 5. Between 0° and 0.5° the curve grows exponentially until an elbow point. This elbow point accords with the maximum number of fixations (Figure 4) for each time window [35]. However, this inflexion point is more difficult to detect as the time window increases. The percentage of points inside a fixation decreases as the time window lengthens but increases with the increment in the dispersion threshold.

**Figure 6:**
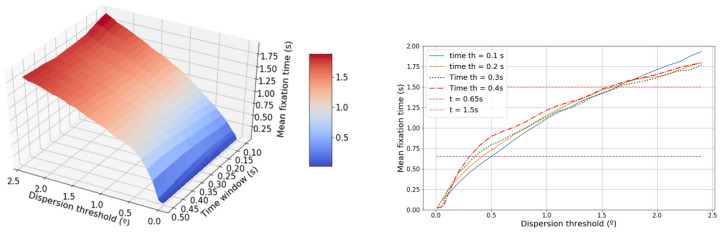
The left-hand graphic shows the evolution of the mean fixation time in terms of the dispersion threshold and the time window. The right-hand graphic shows the projected dispersion threshold.

Figure 6 shows that the mean fixation time depends linearly on the dispersion threshold and does not depend on the time window for the plotted region. The higher is the dispersion threshold the higher is the mean time fixation. A mean time of 1.5 s is achieved for a dispersion threshold of $1.5^{\circ }$, whereas, for a value of $0.5^{\circ }$, a mean time of 0.65 s is achieved.

Taking these results into account and applying the rules established in Table 1, the optimum set of parameters can be inferred from these computed featuresThe rules derived from the features, number of fixations, percentage of points classified as a fixation, and mean fixation time, specify a region of parameters (shown in Table 2). Moreover, based on our optimization strategy, the percentage of points classified as fixations should be as high as possible; this is achieved for a time window of 0.1 s and a dispersion threshold of $1.6^{\circ }$, with a value of 90.43%.

**Figure 7:**
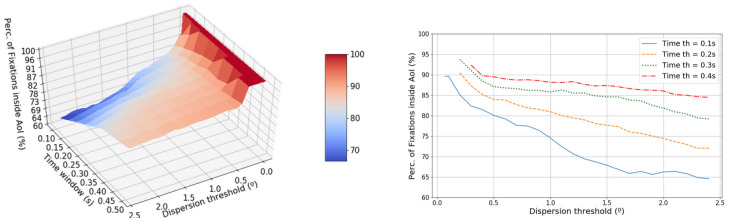
The left-hand graphic shows the evolution of the percentage of fixations inside Area of Interest (AoI) in terms of the dispersion threshold and the time window. The right-hand graphic shows the projected dispersion threshold.

The feature percentage of points inside AoIs was computed and added to the results. As can be seen in Figure 7, the percentage of points inside AoIs has a high degree of dependency on the time window value, as it varies from 76% with 0.1 s to 91% with 0.4 s, maintaining the dispersion threshold in $1^{\circ }$. A flat region is demonstrated for time windows above 0.2–0.50 s (Figure 7) and a dispersion threshold between 0.5–1.2°. This stability is not seen for time windows from 0.1–0.2 s which have a decreasing trend. As the dispersion threshold parameter increases from points higher than 1.3–1.5°, the feature value decreases to zero.

**Figure 8:**
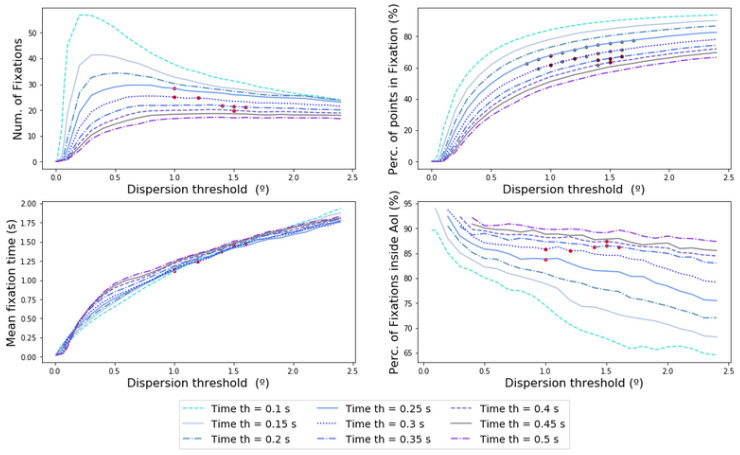
The four different features used to calibrate a dispersion-threshold identification (I-DT) algorithm with the points that fulfill all the conditions established (red points) in terms of the dispersion threshold for different time windows.

The established criterion of stability for the feature percentage of fixations inside AoIs was added to the results of Table 2. A final acceptable set of points that fulfill all the conditions of Table 1 was obtained and is shown at Figure 8.

Table 3 shows the values of the red points of Figure 8. Only four time windows fulfill the conditions imposed by the Table 1. Points where the time window is below 0.25 s are excluded due to high variability in the feature percentage of fixations inside the AoIs. Longer time windows, such as 0.45 s and 0.5 s, are not optimum because the elbow point has not been reached yet. Dispersion points below $1^{\circ }$ and higher than $1.6^{\circ }$ were also discarded, due to the instability of the percentage of fixations inside these AoIs and due to their large mean fixation time values. The results showed a positive correlation between the optimum dispersion points and the optimum time window: When the time window is larger, the dispersion threshold also increases. Following the criteria that the percentage of points classified as fixations should be as high as possible, the highest value found was 67.82%, for a time window of 0.25 s and a dispersion threshold of $1^{\circ }$.

## 4. Discussion

The purpose of this study is to develop an I-DT fixation algorithm to be used in a VR-centered experiment and obtain optimum thresholds for the algorithm in a 3D IVE. The results can be discussed on four levels: (1) the novelty of the algorithm used, (2) the optimum thresholds obtained; (3) comparisons with previous studies; and (4) the calibration procedure used.

The present study experimentally validated the use of head movements using an I-DT fixation identification algorithm, with an integrated ET sensor in a new generation HMD, the HTC Vive Pro Eye. Moreover, we introduced a new frequency threshold. As can be seen in Figure 3, the frequency of the data in an IVE is not always continuous, and it can suffer from high variability due to the IVE renderization; this needs to be considered in VR-centered eye-tracking research. The algorithm presented is robust in the face of these fluctuations, and rejects possible fixation classifications for points below a certain frequency threshold. In addition, as the maximum frequency of the ET device used was 120 Hz, and acquisition in this relatively simple environment was 44.95 Hz, the complexity of the environment needs to be taken into account as it strongly depends on the GPU hardware used. On the other hand, the I-DT algorithm used in this work is not dependent on the dimensionality of the experiment. It could be used to analyze fixation classification in 2D data. This leads to a more general approach to studying ET data.

The strategy followed to calibrate the I-DT algorithm in this research, was to obtain an agreement between the features computed, following the rules established in Table 1. The analysis showed the dependency of the number, duration and position of each fixation in terms of the I-DT parameters. Based on the criteria established for every feature computed, a final set of parameters for the calibration of the I-DT algorithm in an IVE was achieved. Thresholds between $1^{\circ }$ and $1.6^{\circ }$ for dispersion and time windows between 0.25 s and 0.4 s are the optimum, where the point that best fits the fixed criteria is $1^{\circ }$ and 0.25 s. It is interesting that the optimum points found followed a proportional relation between the time window and the dispersion threshold. To select concrete values inside this optimum region, the feature that has to be boosted has to be known [35]. For example, with values of $1^{\circ }$ and 0.3 s, more fixations will be obtained, but the percentage of points classified as being inside fixations will be less than the classification achieved for parameter points $1.2^{\circ }$ and 0.3 s. The results showed a parametrical region that might be considered for future studies where the I-DT fixation classification algorithm is used in an IVE. To the best of the authors’ knowledge, these results present the first calibration of a fixation algorithm in VR.

There are many similarities between the results obtained in this work and previous studies. The features examined in this work, obtained using a VR-centered system, follow similar shapes and trends to features computed in world-centered scenarios [35,36], with different numerical values. Blignaut (2009) [35] showed that the number of fixations achieved is higher than 50 for optimum parameters in an experiment of 15 s duration. The mean fixation time achieved in the present study was 0.25 s for a dispersion threshold of $1.45^{\circ }$. This mean time accords with the mean fixation time established in the literature for world-centered experiments, which is between 0.15 s and 0.65 s [18]. However, Shic et al. (2008) [36] obtained a mean fixation time of 1 s for a dispersion threshold of $1^{\circ }$. On the other hand, VR-centered experiments, such as that of Duchowski et al. (2002) [30], identified a total number of fixations between 15–30 for a 44 s experiment and a mean fixation time of 1.9 s. In the present study, the maximum number of fixations obtained was 28 for the optimum set of parameters, for an experiment of less than 60 s duration. The mean fixation time obtained from the optimum parameters was between 1 s and 1.5 s. The mean fixation time is higher in this study, but this might be due to two reasons. The first might be related to the experimental methodology, where the subjects have to look at a fixed AoI for at least 3 s. The second could be that ET in VR manifests some different characteristics in comparison to 2D schema, which accords with the results obtained by Reference [30]. In addition, the linear relationship between the mean fixation time and the dispersion threshold agrees with the findings of Reference [35,36]. Thus, the results suggested that the number of fixations is lower, and mean fixation time is higher, in VR-centered than world-centered experiments.

The present work used a guided experiment which indicated where the subject had to look every time. However the VR design could introduce bias in the subject’s gaze due the used colors and numeration, which is a limitation of the present study. Previous studies, such as Reference [30,32], also used this type guided experiment to calibrate their own ET algorithms. This methodology allows the researcher to identify what the subjects have been looking at, at any particular time. This is a key point because this methodology allows the researcher to know and define AoIs and record different features to enable spatial and temporal comparisons between subjects. This comparison is not possible in a free-experimentation task because this does not allow a comparative analysis of AoIs between subjects. In the present study the features which can be computed in guided and free tasks, such as number of fixations, gave us preliminary knowledge of the region of agreement of the parameters. The results show dispersion thresholds of $0.3^{\circ }$ to $1.6^{\circ }$, and time windows from 0.1 s to 0.45 s, where the most suitable set of parameters was found for $1.6^{\circ }$ and 0.1 s. This parametrical region accords with the results achieved by Blignaut (2009) [35], an experiment developed with a free ET task. When the feature specifically assigned to measure the guided task, the percentage of fixations inside the AoI, was used, the optimum parametrical region was reduced, which provided more accurate results. The results obtained reduced the time window parameter to the interval 0.25 s to 0.4 s, and the dispersion threshold to 1–1.6°, the optimum point being 0.25 s and $1^{{}^{\circ}}$. This feature complements and specifies the parametrical research. For this reason, a guided experiment provides features appropriate to calibrate a specific ET algorithm. On the other part, the exposure time of the subject to the VR is low, in order to avoid ocular fatigue. However, it would be interesting to evaluate how this fatigue could affect to the results of the optimum parameters in longer experimentations.

## 5. Conclusions

In conclusion, the present study has demonstrated the implications of using an I-DT algorithm in an IVE, which includes some key points as the head movement of the subjects, previously presented by Reference [30]. Moreover, a new frequency threshold is introduced in order to avoid the variation of the frequency which comes from the IVE. The algorithm presented is robust in the face of these fluctuations, and rejects possible fixation classifications for points below a certain frequency threshold. Four different features were used, as no definitive feature exists for modeling visual attention through ET fixation identification algorithms. Different conditions were established for each feature, the optimum thresholds being those that simultaneously accomplish all the conditions of the features. This ends up with a set of parameters which are between $1^{\circ }$ and $1.6^{\circ }$ for dispersion and time windows between 0.25 s and 0.4 s. However, the point that best fits the fixed criteria is $1^{\circ }$ and 0.25 s. We presented a simple case of a guided task, as the experiment did not attempt navigation. Future work will be needed to address how a navigation task influences the calibration. This work established rules to calibrate an ET algorithm; these could be modified based on experiments undertaken and the objectives of the studies. The recent technological developments in VR and ET open a huge new research field combining both technologies. It could mean a breakthrough in the analysis of human behavior in controlled experimental set-ups using immersive VR. The analysis presented, the type of methodology, and the criteria used in this work, provide a useful guide for future research in the use of ET for fixation classification studies in IVE. Furthermore, this research presents a novel I-DT algorithm adapted to a VR-centered ET paradigm, and some innovations, such as frequency acquisition and the use of 3D head movement for the I-DT algorithm. The algorithm was calibrated and obtained a final set of optimized thresholds, which might be used as a tool in future research analyzing gaze patterns in HMDs.

## Figures and Tables

**Table 1:** Calibration criteria of the features.

Measure	Criterium
Number of fixations	After the maximum fixation number
Percentage of points classified inside a fixation	After elbow point and as high as possible
Mean fixation time	Lower than certain predefined time
Percentage of fixations inside AoI	Stable region

**Table 2:** Optimum parameters using the criteria of number of fixations and mean time fixation.

Parameters	Values							
**Dispersion Th** **(°)**	0.3–1.6	0.5–1.5	0.6–1.6	0.8–1.7	0.9–1.6	1.4–1.6	1.4–1.5	1.4–1.5
**Time window** **(s)**	0.1	0.15	0.2	0.25	0.3	0.35	0.4	0.45

**Table 3:** Optimum parameters using the criteria from number of fixations, mean time fixation, and percentage of fixations inside AoI.

Parameters	Values			
**Dispersion Th** **(°)**	1	1 and 1.2	1.4–1.6	1.5
**Time window** **(s)**	0.25	0.3	0.35	0.4

## Supplementary Materials

The I-DT algorithm in VR-centred system is implemented in the following link https://github.com/ASAPLableni/VR-centred_I-DT_algorithm.
